# Evaluating medical education regulation changes in Brazil: workforce impact

**DOI:** 10.1186/s12960-021-00580-5

**Published:** 2021-03-16

**Authors:** Alexandre Medeiros Figueiredo, Danette Waller McKinley, Adriano Massuda, George Dantas Azevedo

**Affiliations:** 1 Health Sciences Postgraduate Program, Universidade Federal do Rio Grande do Norte, Natal, Brazil; 2 Department of Health Promotion, Federal University of Paraíba, Campus I, Jardim Universitário, S/N, Castelo Branco, João Pessoa, PB Brazil; 3 Research and Data Resources, FAIMER, 3624 Market Street, Philadelphia, PA 19104 USA; 4 School of Business Administration, Fundação Getulio Vargas (FGV EAESP), Av. 9 de julho, 2029, Bela Vista, São Paulo, SP 01313-902 Brazil; 5 Multicampi School of Medical Sciences, Federal University of Rio Grande do Norte, Av. Cel. Martiniano, 541, Caicó, RN 59300-000 Brazil

**Keywords:** Medical education, Medically underserved area, Health workforce

## Abstract

**Background:**

Shortages and inequitable distribution of physicians is an obstacle to move towards Universal Health Coverage, especially in low-income and middle-income countries. In Brazil, expansion of medical school enrollment, curricula changes and recruitment programs were established to increase the number of physicians in underserved areas. This study seeks to analyze the impact of these measures in reduce inequities in access to medical education and physicians’ distribution.

**Methods:**

This is an observational study that analyzes changes in the number of undergraduate medical places and number of physicians per inhabitants in different areas in Brazil between the years 2010 and 2018. Data regarding the number of undergraduate medical places, number and the practice location of physicians were obtained in public databases. Municipalities with less than 20,000 inhabitants were considered underserved areas. Data regarding access to antenatal visits were analyzed as a proxy for impact in access to healthcare.

**Results:**

From 2010 to 2018, 19,519 new medical undergraduate places were created which represents an increase of 120.2%. The increase in the number of physicians engaged in the workforce throughout the period was 113,702 physicians, 74,771 of these physicians in the Unified Health System. The greatest increase in the physicians per 1000 inhabitants ratio in the municipalities with the smallest population, the lowest Gross Domestic Product per capita and in those located in the states with the lowest concentration of physicians occurred in the 2013–2015 period. Increase in physician supply improved access to antenatal care.

**Conclusions:**

There was an expansion in the number of undergraduate medical places and medical workforce in all groups of municipalities assessed in Brazil. Medical undergraduate places expansion in the federal public schools was more efficient to reduce regional inequities in access to medical education than private sector expansion. The recruitment component of More Doctors for Brazil Program demonstrated effectiveness to increase the number of physicians in underserved areas. Our results indicate the importance of public policies to face inequities in access to medical education and physician shortages and the necessity of continuous assessment during the period of implementation, especially in the context of political and economic changes.

## Background

The availability of adequate health workforce to meet health needs is an important challenge for health systems to move towards Universal Health Coverage (UHC) [1–3]. Shortages and inequitable distribution of physicians is a global phenomenal that mainly affects low-income and middle-income countries [1–4]. In most countries, the health workforce is concentrated in larger and developed cities. Labor market factors such as better employment opportunities, practicing conditions and training opportunities make these regions more attractive [2, 4]. Even countries with well-established health systems have difficulty in attracting and retaining professionals in rural and underserved areas [5, 6]. This complex problem is influenced by economic, social and cultural factors [4]. Consistent and long-term public policies are necessary to promote the supply of physicians and their retention in underserved areas.

The main strategy to reach an adequate number of physicians in order to meet health needs has been the expansion of medical school enrollment [2, 4]. While essential, expansion alone is inadequate to guarantee the attraction and retention of physicians in underserved areas [4]. Interventions including professional regulation, financial incentives, and support activities for the education and work sectors are described in the literature as additional strategies to increase the number of physicians in underserved areas [7, 8]. Educational policies have proven to be effective and these policies include specific admission programs for students from underserved areas, expansion of undergraduate and postgraduate (residency) programs in regions with a low ratio of physicians per inhabitant, development of learning experiences in rural areas and curricula that prioritize primary health care (PHC) competencies [7, 8].

**Fig. 1:**
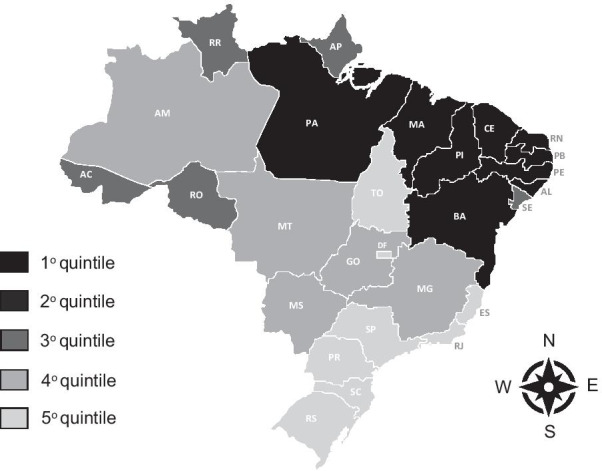
Gross domestic product per state, Brazil, 2010

In Brazil, the Unified Health System (SUS) was established by the 1988 Constitution based on the principles of health as a citizen’s right and the state’s duty. The SUS aims to provide comprehensive health care through decentralized management and provision of health services [9]. Since the establishment of SUS inequalities in the number and regional distribution of physicians persisted as one of the main obstacles to universal and equitable access to healthcare [9, 10]. The areas with shortage of physicians are concentrated in rural and remote areas, mainly in the North and Northeast regions, which are the regions with the lowest economic development (Fig. 1) [9].

The chronic underfunding of SUS and the high percentage of private health spending in Brazil aggravated the inequality in the distribution of these professionals [11]. Several regulatory mechanisms and programs to increase the number of physicians and improve their distribution have been implemented by the Brazilian Federal Government [12, 13]. Proposals for recruiting physicians to practice in PHC in underserved areas, such as the Program for the Interiorization of Health Work (Programa de Interiorização do Trabalho em Saúde, PITS-2001-2003) and the Program for Valuing Primary Care Professionals (Programa de Valorização dos Profissionais da Atenção Básica, PROVAB-2011) were also implemented nationally [12].

**Fig. 2:**
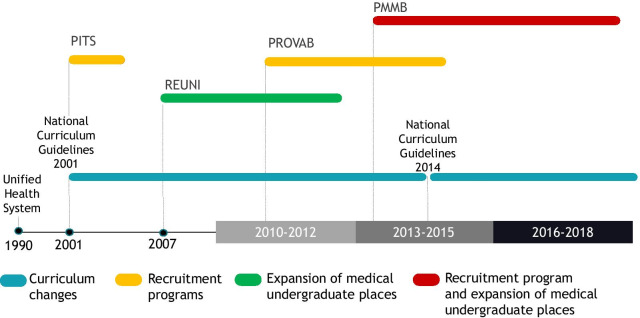
Timeline of policies to promote the supply of physicians in Brazil

In parallel, educational policies were implemented. In 2001, publication of the National Curricular Guidelines was a milestone for emphasizing general training and expansion of practice scenarios to prioritize PHC settings [14]. In addition, there was an expansion of access to higher education in Brazil through the Program of Support for the Restructuration and Expansion of Federal Universities (Programa de Apoio a Planos de Reestruturação e Expansão das Universidades Federais, REUNI), created in 2007, directing the expansion of medical schools to municipalities located in regions with a lower physician ratio per inhabitant [2, 13]. We document the timeline of policies in Fig. 2.

These policies, however, were not sufficient to address physician shortages especially in PHC [9, 13]. In 2013, the More Doctors for Brazil Program (Programa Mais Médicos para o Brasil, PMMB) was created to guarantee a medical workforce adequate to the health needs of the Brazilian population, reduce regional inequalities and increase the number of physicians practicing in PHC [15]. This program was defined as the main strategy to reach the proportion of 2.7 physicians per 1000 inhabitants by 2026 [13]. To achieve this result, the PMMB employs the following strategies: recruiting physicians to practice in PHC, expanding the number of undergraduate and medical residency enrollments and promoting curricula changes [13, 15].

Both Brazilian-educated and foreign-educated physicians are eligible under recruitment regulations [15]. The PMMB offers financial incentives with medical contracts of up to 6 years, grants for housing and food and a support program developed by public education institutions [9, 13, 15]. Professional regulation was modified to allow physicians with foreign undergraduate diplomas to be able to practice medicine in the PMMB without need to validate their diplomas via exams [15]. This measure allowed the incorporation of approximately 12,000 foreign physicians in PHC, made it possible to substantially increase the coverage of care in the country, especially in underserved areas [9]. The engagement of foreign physicians in the PMMB began in 2013 and increased until the end of 2015 [9]. Then, there was a gradual decline in foreign physician engagement that was highlighted in December 2018 when cooperation with the Cuban government ended [16].

The medical education component of the PMMB is the mainly strategy to increase physician’s supply in Brazil [17]. PMMB set the goal of creating 11,500 new undergraduate places through 2017 [17]. The PMMB established a new regulatory framework for opening public and private medical schools, prioritizing the allocation of these schools in areas of physician shortages [13, 15]. The opening of new private schools was regulated by a new model in which the federal government defines a municipality in an underserved area for the establishment of the medical school, then a public call is made to choose the private institution responsible to implement the program [13, 15].

Additionaly, a new undergraduate medical curriculum was developed to strengthen PHC training [13, 18]. This included changes in the process of evaluating and accreditation of medical schools by adapting the evaluation tool for undergraduate education in the National Higher Education Assessment System (Sistema Nacional de Avaliação da Educação Superior; Sinaes). The new medical curriculum guidelines and objectives of the PMMB were used to implement these changes [13, 19]. This regulatory framework was expanded to award greater value to institutions integrated with the local health system, those with greater capacity to offer medical residency programs in priority specialties and those with greater social accountability and adaptation to local social health needs [13, 19]. Finally, the PMMB established a new paradigm for regulation of residency in Brazil with inclusion of an initial year of training in Family and Community Medicine for residency programs of most medical specialties [13, 15]. However, the latter strategy was never implemented.

This study analyzes whether changes in medical education regulation and physician recruitment and retention between 2010 and 2018 met the objectives of increasing the number of medical undergraduate places, number of physicians and reduction in inequalities in the access to medical education and in physician’s distribution.

## Methods

This is an observational study that analyzes changes in the number of medical undergraduate places and the increase in the number of physicians in Brazil between the years 2010 and 2018. In order to assess the changes in medical undergraduate places, the absolute number of places and the ratio of undergraduate places per 10,000 inhabitants were analyzed. The analysis of the evolution in the number of physicians in Brazil was carried out based on the total number of physicians and the number of physicians practicing in the SUS, aiming to understand the impact of the increase in undergraduate places and recruitment programs on the public and private workforce. In both cases, the ratio of physician per 1000 inhabitants was calculated considering the total number of physicians and the number of physicians practicing in SUS.

Data regarding the number of medical undergraduate places were obtained from the database on undergraduate education of the Higher Education Census of the National Institute of Studies and Research Anísio Teixeira (Instituto Nacional de Estudos e Pesquisas Anísio Teixeira, INEP) [20]. Data concerning the number and the practice location of physicians were obtained in January of 2020 from the National Register of Healthcare Establishments (Cadastro Nacional de Estabelecimentos de Saúde, CNES) [21]. This database registers all public and private health care facilities in Brazil and its health professionals monthly [21]. We extracted data for December of each year to calculate the study variables. The population data used in the study was based on population estimates developed by the Brazilian Institute of Geography and Statistics (Instituto Brasileiro de Geografia e Estatística, IBGE) [22].

The values found in 2009 were used as a baseline for the analysis of changes in the period. As described in the background, the period under analysis was characterized by the implementation of interventions with different beginnings and durations, as well as changes in federal government. Thus, we carried out the analysis in three distinct periods.

The first time period (2010–2012) represents the period prior to the PMMB, the second (2013–2015) represents the implementation of the program under the Dilma Rousseff administration and the third period (2016–2018), corresponded to a later phase of the PMMB during the Michel Temer administration. The variation in the number of physicians and the number of undergraduate places was calculated using data from the last year of each 3-year period. Thus, the number of medical undergraduate places created and the number of physicians engaged in the medical workforce in the first three years were obtained from the difference obtained by comparing the difference between the 2010 and 2012 data. The values for the second 3-years period were calculated from the difference between the values of 2013 and 2015 and the values of the third triennium of the difference between 2016 and 2018.

In order to identify changes in the distribution of new medical undergraduate medical places and the engagement of physician in the workforce, data were aggregated by macro-regions and by characteristics of the municipalities in 2009 (baseline) such as: municipal population size, physician inhabitant rate in the state and gross domestic product (GDP) per capita [23]. The categorization related to physician per inhabitant ratio in the state was obtained using the mean of the values derived from the physician per inhabitant ratio in each state of the federation in 2009. Data on medical undergraduate places were also aggregated by medical school ownership into: private, federal public schools, state public schools and municipal public schools. Schools classified in the Higher Education Census as a “special” ownership category were categorized as municipal, as they are governed by municipal laws and have approval flow from the State Education Councils.

The absolute variations in the medical undergraduate places ratio per 10,000 inhabitants and physicians per 1000 inhabitants for the entire period were calculated from the difference between the values found in 2009 and 2018. The relative variations, in turn, were calculated using the formula:

$$\frac{\mathrm{Value}\;\mathrm{in}\;2018}{\mathrm{Value}\;\mathrm{in}\;2009}-1\times100$$

Finally, data from the National Information System on Live Births (Sistema de informações de Nascidos Vivos, SINASC) were used to assess variations in access to antenatal visits during the study [24]. We analyze differences in percentage of pregnant women with more than 6 antenatal care visits between the years 2010 and 2018 in each state as a proxy of increase in healthcare access. We conducted Friedman test to determine whether there were statistically significant differences.

## Results

From 2010 to 2018, 19,519 new medical undergraduate places were created (variation from 16,236 to 35,755), which represents an increase of 120.2%. Of these, 18,014 (92.2%) undergraduate places were created after the PMMB implementation (Table 1).

**Table 1:** Evolution of absolute number of medical undergraduate places and ratio per inhabitants

Municipality characteristics	2009		2018		Increase in medical undergraduate places per 10,000 inhabitants from 2010 to 2018	
	Medical undergraduate medical places		Medical undergraduate medical places			
	Places	Ratio per 10,000 inhabitants	Places	Ratio per 10,000 inhabitants	Absolute increase	Relative increase (%)
Macro-region						
Midwest	953	0.69	2759	1.72	1.03	150.08
Northeast	3318	0.62	8362	1.47	0.85	137.95
North	1293	0.84	2701	1.49	0.65	76.76
Southeast	8448	1.04	16723	1.91	0.86	82.61
South	2224	0.80	5210	1.75	0.95	118.24
*Population*						
Up to 100,000 inhabitants	1305	0.15	5177	0.55	0.40	266.92
Between 100,001 and 500,000 inhabitants	5855	1.25	13812	2.65	1.40	111.62
Above 500,000 inhabitants	9076	1.58	16766	2.71	1.13	71.22
*Physician inhabitant ratio in 2009*						
Up to 01 physician per 1000 inhabitants	2677	0.58	6477	1.29	0.71	123.32
Between 01 and 1.5 physicians per 1000 inhabitants	3317	0.74	9016	1.83	1.09	146.47
Above 1.5 physicians per 1000 inhabitants	10242	1.02	20262	1.86	0.84	82.35
*GDP per capita in 2009*						
Below the national average	1383	0.19	4534	0.58	0.39	209.05
Above the national average	14853	1.26	31221	2.40	1.14	90.02
*Medical school ownership*						
State public schools	1712	0.09	2362	0.11	0.02	26.73
Federal public schools	4561	0.24	6663	0.32	0.08	34.18
Municipal public schools	266	0.01	1557	0.07	0.06	437.64
Private	9697	0.51	25173	1.21	0.70	138.44

The Midwest macro-region showed the highest relative growth in the medical undergraduate places ratio per 10,000 inhabitants (150.08%), followed by the Northeast (137.95%) and South (118.24%) macro-regions. When observing the absolute growth in the medical undergraduate places per 10,000 inhabitants, the largest increases occurred in the Midwest (1.03 medical undergraduate places per 10,000 inhabitants), South macro-region (0.95 medical undergraduate places per 10,000 inhabitants) and Southeast macro-region (0.86 medical undergraduate places per 10,000 inhabitants). As noted, the North and Northeast macro-regions remained the regions with the lowest medical undergraduate places ratio per inhabitant. States with an intermediate situation in relation of physician’s shortages had the largest absolute and relative increase in the medical undergraduate place’s ratio per inhabitant (Table 1). The greatest relative increase in the medical undergraduate places ratio per inhabitant occurred in municipalities with GDP below the national average in 2009. However, the absolute increase in the undergraduate medical places ratio per inhabitants occurred in the wealthiest municipalities.

In comparing the three time periods, it is observed that from 2010 to 2012, 1,505 medical undergraduate places were created (7.7% of the total), 6791 (34.8% of the total) in the triennium 2013–2015, and 11,223 (57.5% of the total) in the triennium 2016–2018 (Table 2). In these last three years, 5391 (48.0%) medical undergraduate places were opened in the Southeast region (Table 2). In addition, there was greater expansion in municipalities with more than 500,000 inhabitants and in states that already had more medical undergraduate places per 10,000 inhabitants (Table 2).

**Table 2:** Medical undergraduate places created and ratio per 10,000 inhabitants per triennium

	Medical undergraduate places increases					
Municipality characteristics	2010–2012		2013–2015		2016–2018	
	Absolute	Ratio per 10,000 inhabitants	Absolute	Ratio per 10,000 inhabitants	Absolute	Ratio per 10,000 inhabitants
*Macro-region*						
Midwest	97	0.04	1247	0.76	462	0.23
Northeast	553	0.10	1638	0.26	2853	0.50
North	105	0.01	378	0.16	925	0.47
Southeast	370	0.04	2514	0.24	5391	0.58
South	380	0.14	1014	0.30	1592	0.51
*Population*						
Up to 100,000 inhabitants	345	0.04	1685	0.17	1842	0.19
Between 100,001 and 500,000 inhabitants	750	0.14	2826	0.48	4381	0.78
Above 500,000 inhabitants	410	0.06	2280	0.30	5000	0.77
*Physician inhabitant ratio in 2009*						
Up to 01 physician per 1000 inhabitants	358	0.07	1089	0.19	2353	0.46
Between 01 and 1.5 physicians per 1000 inhabitants	649	0.13	2487	0.47	2563	0.49
Above 1.5 physicians per 1000 inhabitants	498	0.04	3215	0.25	6307	0.55
*GDP per capita in 2009*						
Below the national average	245	0.03	1326	0.16	1580	0.20
Above the national average	1260	0.08	5465	0.36	9643	0.70
*Medical school ownership*						
State public schools	265	0.01	201	0.00	184	0.01
Federal public schools	278	0.01	1447	0.06	377	0.01
Municipal public schools	154	0.01	477	0.02	660	003
Private	808	0.04	4666	0.20	10002	047

Regarding the medical school ownership, 15,476 medical undergraduate places were created in private institutions, 10,002 (64.63%) of which were created in the 2016–2018 triennium (Table 2). The greatest public expansion took place within the scope of federal public medical schools with the creation of 2102 places, of which 1447 (68.84%) in the 2013–2015 period. In municipal public medical schools, 1291 places (51.12%) were created in the 2016–2018 period (Table 2).

**Fig. 3:**
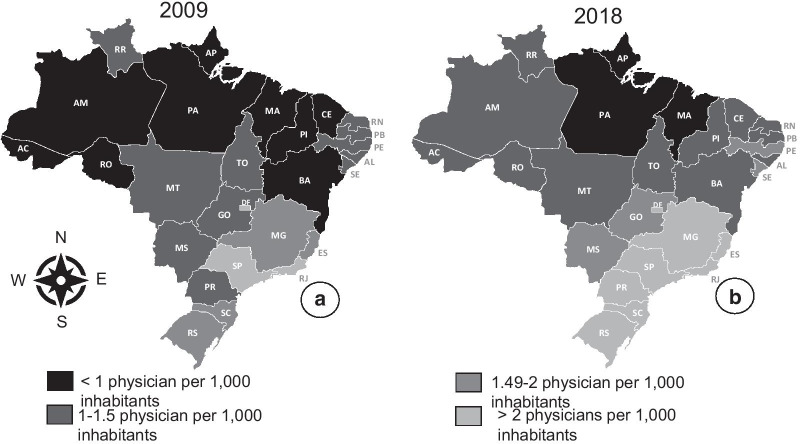
Physician per inhabitant ratio by state, 2009 and 2018, Brazil

The increase in the number of physicians engaged in the workforce throughout the period was 113,702 physicians, 74,771 of these physicians in the SUS (Table 3). Figure 3 shows the physician per inhabitant ratio by state in 2009 (baseline) and 2018.

**Table 3:** Evolution of absolute number of physicians (total and SUS) and physicians ratio per 1000 inhabitants

Municipality characteristics	Physicians practicing in SUS				Total of physicians			
	2009		2018		2009		2018	
	Absolute number	Physician per 1000 inhabitants ratio	Absolute number	Physician per 1000 inhabitants ratio	Absolute number	Physician per 1000 inhabitants ratio	Absolute number	Physician per 1000 inhabitants ratio
*Macro-region*								
Midwest	16342	1.18	22687	1.41	20975	1.51	31084	1.93
Northeast	42947	0.80	59614	1.05	50962	0.95	72154	1.27
North	10838	0.70	15525	0.85	12146	0.79	18430	1.01
Southeast	111575	1.38	144515	1.65	155142	1.92	210456	2.40
South	33682	1.22	47814	1.61	43386	1.57	64189	2.16
*Population*								
Up to 20,000 inhabitants	16919	0.51	20821	0.60	17398	0.52	22095	0.63
Between 20,001 and 50,000 inhabitants	20544	0.64	26077	0.75	22487	0.70	30450	0.87
Between 50,001 and 100,000 inhabitants	19638	0.88	26979	1.09	23102	1.04	33808	1.37
Between 100,001 and 500,000 inhabitants	59139	1.26	81921	1.57	77202	1.65	112150	2.15
Above 500,000 inhabitants	99144	1.73	134357	2.17	142422	2.48	197810	3.20
*Physician inhabitant ratio in 2009*								
Up to 01 physician per 1000 inhabitants	32288	0.70	44495	0.89	37637	0.81	54455	1.08
Between 1.0 and 1.5 physicians per 1000 inhabitants	44781	1.00	63425	1.29	54957	1.23	79986	1.63
Above 1.5 physicians per 1000 inhabitants	138315	1.38	182235	1.67	190017	1.89	261872	2.40
*GDP per capita in 2009*								
Below the national average	42059	0.57	54506	0.70	46201	0.63	62965	0.80
Above the national average	173325	1.47	235,649	1.81	236410	2.01	333348	2.56

**Fig. 4:**
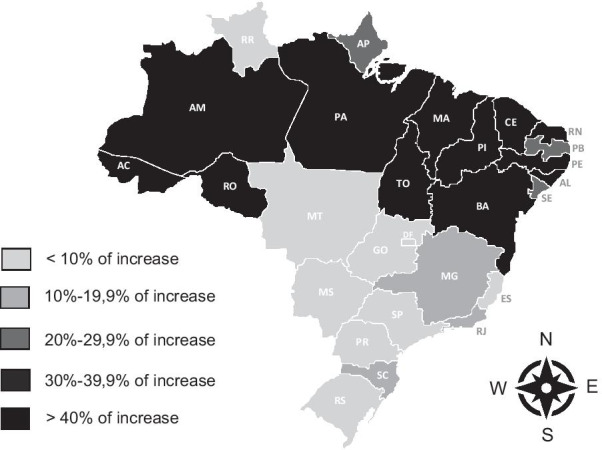
Increase in the percentage of pregnant women with > 6 antenatal visits between 2010 and 2018

The municipalities with the largest population and with the highest GDP per capita had the highest physician per 1000 inhabitants ratio in 2018 (Table 3). These data reveal the importance of socioeconomic factors as determinants in the distribution of physicians. In municipalities below 20,000 inhabitants, almost the entire medical workforce is guaranteed by SUS.

The medical workforce increased by 23,833 doctors in the 2010–2012 period, 45,666 doctors in the 2013–2015 period and 44,203 doctors in the 2016–2018 period (Table 4). We observed that the increase in the workforce at SUS was 17,817 physicians (74.76% of total) in the period 2010–2012, 32,172 physicians (70.45% of total) in the period 2013–2015 and 24,838 (56.19% of total) in 2016–2018 (Table 5).

**Table 4:** Variation of physicians in the workforce and ratio per 1000 inhabitants per triennium

Municipality characteristics	2010–2012		2013–2015		2016–2018		2010–2018	
	Absolute number	Physician per inhabitants ratio	Absolute number	Physician per inhabitants ratio	Absolute number	Physician per inhabitants ratio	Absolute increase in the physician per inhabitants ratio	Relative increase in the physician per inhabitants ratio (%)
*Macro-region*								
Midwest	2198	0.10	3721	0.14	4190	0.19	0.42	28.01
Northeast	4140	0.07	9968	0.13	7084	0.12	0.32	33.68
North	914	0.01	3647	0.16	1723	0.06	0.22	28.40
Southeast	12546	0.14	20140	0.13	22628	0.21	0.48	25.14
South	4035	0.14	8190	0.19	8578	0.25	0.59	37.83
*Population*								
Up to 20,000 inhabitants	− 408	− 0.01	4060	0.10	1045	0.03	0.11	20.95
Between 20,001 and 50,000 inhabitants	680	0.01	4915	0.11	2368	0.06	0.17	23.90
Between 50,001 and 100,000 inhabitants	1337	0.03	5163	0.15	4206	0.14	0.33	31.74
Between 100,001 and 500,000 inhabitants	7893	0.14	12939	0.15	14116	0.21	0.50	30.31
Above 500,000 inhabitants	14331	0.23	18589	0.17	22468	0.30	0.71	28.73
*Physician inhabitant ratio in 2009*								
Up to 01 physician per 1000 inhabitants	2793	0.05	8766	0.13	5259	0.09	0.27	33.54
Between 01 and 1.5 physicians per 1000 inhabitants	4791	0.08	10460	0.15	9778	0.17	0.39	31.98
Above 1.5 physicians per 1000 inhabitants	16249	0.15	26440	0.15	29166	0.22	0.51	27.03
*GDP per capita in 2009*								
Below the national average	1840	0.02	10337	0.10	4587	0.05	0.18	28.47
Above the national average	21993	0.15	35329	0.16	39616	0.25	0.55	27.47

**Table 5:** Increase of physicians in SUS and ratio per 1000 inhabitants per triennium

Municipality characteristics	2010–2012		2013–2015		2016–2018		2010–2018	
	Absolute number	Physician per 1000 inhabitants ratio	Absolute number	Physician per 1000 inhabitants ratio	Absolute number		Absolute number in the physician per inhabitants ratio	Relative increase in the physician per inhabitants ratio (%)
*Macro-region*								
Midwest	1125	0.03	2188	0.06	3032	0.14	0.23	19.92
Northeast	3336	0.06	8595	0.11	4736	0.08	0.25	31.06
North	438	− 0.01	3257	0.14	992	0.02	0.15	21.21
Southeast	10072	0.11	12073	0.07	10795	0.09	0.27	19.49
South	2846	0.10	6003	0.14	5283	0.15	0.39	32.25
*Population*								
Up to 20,000 inhabitants	− 603	− 0.02	3842	0.09	663	0.02	0.09	17.20
Between 20,001 and 50,000 inhabitants	98	− 0.01	4258	0.09	1177	0.02	0.10	16.14
Between 50,001 and 100,000 inhabitants	770	0.01	3978	0.11	2593	0.08	0.21	23.68
Between 100,001 and 500,000 inhabitants	5507	0.09	9818	0.11	7457	0.10	0.31	24.26
Above 500,000 inhabitants	12045	0.20	10220	0.08	12948	0.17	0.44	25.61
*Physician inhabitant ratio in 2009*								
Up to 01 physician per 1000 inhabitants	1927	0.03	7266	0.11	3014	0.05	0.19	27.20
Between 01 and 1.5 physicians per 1000 inhabitants	3500	0.06	8648	0.12	6496	0.11	0.29	28.43
Above 1.5 physicians per 1000 inhabitants	12390	0.11	16202	0.08	15328	0.11	0.29	21.44
*GDP per capita in 2009*								
Below the national average	706	0.01	9114	0.09	2627	0.03	0.13	22.17
Above the national average	17111	0.12	23002	0.09	22211	0.13	0.34	22.90

The two macro-regions with the highest relative growth in the physicians per 1000 inhabitants ratio were the South and Northeast regions, respectively. However, the largest absolute increases occurred in the South and Southeast (Tables 4 and 5). The increase in the physicians per 1000 inhabitants ratio occurred in the largest and wealthiest municipalities in the three periods analyzed. The greatest increase in the physicians per 1000 inhabitants ratio in the municipalities with the smallest population, the lowest GDP per capita and in those located in the states with the lowest concentration of physicians occurred in the 2013–2015 period (Tables 4 and 5).

The percentage of pregnant women with more than 6 antenatal care visits was 60.6% in 2010 and 70.8% in 2018 in Brazil, an increase of 17%. This increase varied between 2.4% and 83.9% among Brazilian states. The greatest increases occurred in the poorest states and with the worst physician per inhabitant ratios. Differences in the percentage of pregnant women with more than 6 antenatal care visits between 2010 and 2018 in Brazilian states were statistically significant (p < 0.001) (Fig. 4).

## Discussion

From 2010 to 2018 there was an expansion in the medical workforce in all groups of municipalities assessed in Brazil. This increase can be attributed to the increase in the number of medical undergraduate places as well as to the recruitment of physicians trained abroad through the PMMB. As undergraduate training takes six years in Brazil, the results of the expansion of undergraduate places in the physician workforce is partial and reflect only the increase in enrollments that occurred until 2012.

The expansion planned by the PMMB was to create 11,500 new medical undergraduate places by 2017, reaching a ratio of 1.34 medical undergraduate places per 10,000 inhabitants [17]. According to data from the Higher Education Census, this target was reached in 2017. It was also noted that, in addition to the PMMB goal, just over 7500 additional medical undergraduate places were created by 2018.

The 2013–2015 period presented the greatest reductions in regional inequalities in the distribution of medical undergraduate places. In this triennium there was the greatest expansion of medical undergraduate places in the federal public schools, mostly in the establishment of new campuses in municipalities in Brazil´s countryside [2]. A previous analysis demonstrated that municipalities in countryside in which a medical school was established increased the capacity for attracting and retaining physicians as well expanding healthcare services [2]. The Northeast macro-region (the poorest macro-region in Brazil) had the highest increase in medical undergraduate places in public federal schools. These results demonstrate that expansion of federal medical schools was the most effective measure to reduce inequalities in access to medical education. The expansion of medical undergraduate places in private schools was effective to increase the number of medical undergraduate places but did not reduce regional inequalities.

In addition, it is important to consider that the increase in medical undergraduate places in private institutions does not represent an increase in access to medical education for the poorest population living in vulnerable regions due to the tuition and fees. This situation could be an obstacle to reduce healthcare access inequities, since students from vulnerable regions are more likely to practice in these regions after completing their studies [8, 25].

During the period analyzed, there was an expansion of 113,702 physicians in the workforce, which enabled an increase in the physicians per inhabitant’s ratio in Brazil. The 2013–2015 was the period with the greatest increase in the number of doctors due to the incorporation of graduates from Brazilian medical schools and the recruitment of physicians trained outside Brazil [9]. Due to the regulatory framework of the PMMB regarding recruitment, all physicians trained abroad were incorporated to practice exclusively in primary care in the SUS [15]. In the other three-year periods, the incorporation of foreign doctors was small, and the physician supply was predominantly composed of physicians trained in Brazil. It is also observed that the percentage of physicians who were incorporated into the medical workforce in SUS declined over time. This phenomenon of greater incorporation of physicians in the private sector may pose a problem in ensuring a medical workforce in regions with less economic development in the future.

Despite this increase in the physician inhabitant ratio in Brazil, many municipalities still had less than one physician per 1,000 inhabitants. This situation is prevalent in municipalities with less than 50,000 inhabitants, where more than 65 million Brazilians lived in 2018 [22]. It is noteworthy that about 90% of the medical workforce living in municipalities between 20,000 and 50,000 inhabitants were guaranteed by SUS in 2018. This percentage reached 95% in municipalities with less than 20,000 inhabitants. In the 2013–2015 period, there was a substantial expansion in smaller municipalities, and the increase in the physician-inhabitant ratio in these locations were similar to that found in larger municipalities, especially when considering only physicians practicing in the SUS. These results reflect the importance of the PMMB's recruitment component and the government incentives to expand primary care in SUS to reduce inequalities in access to healthcare, although physicians graduated in Brazil and Brazilians trained in other countries have priority in the recruitment program, Cuban physicians, recruited as part of a Brazilian government cooperation with Cuba government, were an important contingent of professionals who practiced in rural and remote areas [9, 26]. This cooperation generated intense debate during its establishment.

The expansion of medical undergraduate places and the increase in the physician ratio per inhabitant show the importance of specific public policies to increase physician supply. Our results demonstrate that the regulatory mechanisms developed to expand medical undergraduate places in areas with the greatest need of physicians were only partially implemented. It is worth noting that only 20% of medical undergraduate places in private institutions were created through the regulatory framework that was defined by the PMMB. Recruitment of physicians from the PMMB has substantially increased the number of physicians practicing in underserved areas during the years 2013 to 2015 [9]. Growth in the 2016–2018 3-year period was lower, which could be due to the economic crisis and the fiscal austerity policies implemented in 2016 that reduced public investment to expand healthcare in underserved areas [11].

This challenge worsened with the end of cooperation with the Cuban government as these physicians represented the largest contingent of physician in the areas of greatest difficulty in supply [9, 16]. In December 2019, the federal government created the Doctors for Brazil Program [27]. This program established a new structure to expand the hiring of physicians in underserved areas and revoked the changes in the training of specialists defined by the PMMB [26]. However, none of the new measures proposed to increase the number of physicians in underserved areas of this program have been implemented.

## Conclusion

The Brazilian experience brings important reflections to the challenge of facing physician shortages. This experience highlights the need for the development of integrated public policies throughout the lifecycle of the health worker (education, recruitment, retention). The implementation of educational policies in Brazil has been influenced by political and economic changes and part of the proposed regulatory mechanisms have not been implemented as planned. This situation demonstrates the importance of evaluating the implementation of public policies to identify problems, new demands, and challenges. The expansion of federal medical schools was effective to reduce inequities in access to medical education and to increase the supply of physicians. Our results indicate that the expansion of medical undergraduate places in the private sector did not meet the regulatory frameworks established in the PMMB and increased regional inequalities. Thus, it is recommended that the established regulatory framework be followed in the future planning of the offer of medical undergraduate places. Data regarding the origin of medical students must be analyzed to guide the formulation of admission programs for students from underserved areas. This is an effective measure to increase physicians supply and could be an important strategy to be included in medical education regulation in Brazil. Expanding public investment in health to expand and maintain SUS health services, especially in municipalities with a shortage of physicians, is another recommended measure to move towards Universal Health Coverage (UHC).

## Data Availability

All data is disponible in public databases.

## Abbreviations

UHC: Universal health coverage
PMMB: More doctors for Brazil Program
PHC: Primary Health Care
SUS: Unified Health System
PITS: Health Work Interiorization Program
REUNI: Restructuring and Expansion Program of Federal Universities
PROVAB: Program for Valuing Primary Care Professionals
SINAES: National Higher Education Assessment System
INEP: National Institute of Studies and Research Anísio Teixeira
CNES: National Register of Healthcare Establishments
IBGE: Brazilian Institute of Geography and Statistics
GDP: Gross domestic product
